# The Diseases of the Bible

**Published:** 1888-03

**Authors:** 


### Reviews of Books.

---

*The Diseases of the Bible.* By SIR RISDON BENNETT, M.D., LL.D., F.R.S. London: THE RELIGIOUS TRACT SOCIETY. 1887.

THIS is one of a series entitled "By-Paths of Bible Knowledge," and is not intended solely for medical men. If it is taken for granted that a "popular" book on the Diseases of the Bible is desirable, Sir Risdon Bennett's little work no doubt answers the purpose fairly well. We are, however, inclined to doubt the wisdom of any such book, and in justification of this feeling Sir Risdon's words in the Introduction may be quoted: "Some of the diseases mentioned in the Bible are simply named, or so briefly described that we can only surmise what they were. And with regard to others it will be well to admit *in limine* that the most learned physicians of the present day find it difficult to identify them with any particular forms of disease with which they are familiar." (p. 11.) Surmises in reference to sacred subjects or matters connected therewith are better avoided. We want firmer ground. Times have changed since Richard Mead wrote his *Medica Sacra*, in the preface to which he says: "I have not writ these essays for the profane or vulgar; but for those only who are well versed, or at least initiated, in theological or medical studies: and for this reason I chose to publish it in Latin."*

Sir Risdon Bennett, like Mead, and Shapter of Exeter in 1834, includes old age amongst the Diseases of the Bible. There is no need for this, as the phenomena of senile decay have been the same from the foundation of the world. Statements about the aphrodisiacal powers of the caper are

* English Edition, translated by Thomas Stack, M.D. 1755.

certainly out of place in a book addressed to general readers.

Of the 91 pages devoted to actual diseases, more than 38 are occupied with a discussion of leprosy and its essential nature and its hygiene. It is difficult to see the use of a popular treatment of this and allied subjects, which can only be satisfactorily discussed by one who has a wide acquaintance with Oriental modes of life and thought and language.

The observations on the "Physical Cause of the Death of Christ" we think entirely out of place. The issues of that awful event do not concern us in their definite material characters, and no good can result from submitting these to critical analysis.

In other respects the book is of interest to doctors, to whom it will reveal much light on matters directly connected with their profession. Much misconception exists about the diseases mentioned in the Bible. Even so scholarly a work as The New Testament Commentary edited by Bishop Ellicott, considers that the death of Herod Agrippa I. was caused by the *morbus pedicularis*. This mistake could not have been made if the writer had asked the opinion of his medical attendant on the passage. Sir Risdon Bennett, as did Mead, attributes the death to perforation of the bowel, caused by mischief set up by intestinal worms.

